# Rural E-commerce development and farmers’ digital credit behavior: Evidence from China family panel studies

**DOI:** 10.1371/journal.pone.0258162

**Published:** 2021-10-15

**Authors:** Gao Yu, Hu Xiang

**Affiliations:** 1 FANLI Department of Business, Nanyang Institute of Technology, Nanyang, China; 2 School of Applied Economics, Renmin University of China, Beijing, China; Institute for Advanced Sustainability Studies, GERMANY

## Abstract

This paper examines the impact of rural e-commerce development on rural households’ digital credit behavior at the micro-level by using a multivariate Probit model and propensity score matching method with rural residents in the China Family Panel Studies (CFPS) database. Specifically, we examine the complementary or substitution relationship between digital credit and traditional bank credit and the impact of participation in e-commerce on the scale of digital credit of rural households. The empirical results show that there is a substitution relationship between digital credit and traditional bank credit. Participation in e-commerce has a positive impact on the scale of digital credit and the full scale of credit obtained by farmers, with an increase of $0.922 million and $37.49 million in the scale of digital credit and real credit received by farmers who participate in e-commerce, respectively, compared with those who do not participate in e-commerce. Further tests revealed that the difference in capital endowment was an essential reason for the disparity in the size of digital credit received among e-commerce farmers.

## I. Introduction

Developing inclusive finance in rural areas has long been the focus and difficulty of research in the field of rural finance. Numerous studies have shown that rural households face a severe shortage of credit supply. The reasons for this include: on the one hand, farmers may play the role of "negative borrowers" due to information asymmetry, collateral constraints, or risk constraints, while higher credit transaction costs may cause some quality borrowers to choose self-rationing and leave the formal credit market [1–3]. On the other hand, the rise in the scale of borrowing by farmers, the increase in social "human costs," and the weakening of solid ties in social relationships make the desire to rely on informal borrowing from friends and relatives to meet financial needs unlikely [4, 5].

In recent years, information technologies such as big data, cloud computing, and artificial intelligence have been interfused with finance, driving the rapid development of fintech and digital finance, which are seen as potentially transformative financial models to solve the problem of complex financing for farmers [6, 7]. In 2015, the Chinese government released the Report on the Plan for Promoting the Development of Inclusive Finance (2016–2020), which states that "all types of inclusive financial service providers should be actively guided to reduce financial transaction costs, extend the service radius, and expand the breadth and depth of inclusive financial services with the help of modern information technology means such as the Internet." Digital credit built on information technology such as big data, cloud computing, and artificial intelligence has gradually broken through the time and space limitations of traditional financial supply. It has unparalleled advantages in improving information asymmetry, increasing service efficiency, reducing transaction costs, and optimizing risk control. Existing studies have found that ICT(Information and Communications Technology) can help reduce operational costs on the supply side and search and switching costs on the demand side, thereby increasing the availability and scale of credit for borrowers [8, 9], and can provide access to financial services for relatively remote, poor and disadvantaged people [10, 11]. At the same time, the development of information and communication technologies such as the Internet and big data and their application in the financial sector have a catalytic effect on rural financial innovation and financial supply, which can reduce financial exclusion and promote the development of inclusive finance in rural areas [12].

Digital credit in this paper refers to loans from financial institutions obtained by farmers through Internet channels rather than offline channels, which include online loans obtained from e-commerce platforms and online loans from banks. It does not have offline credit from traditional banks (referring to farmers’ access to conventional offline loans from formal financial institutions, such as rural commercial banks, agricultural banks, and village banks, etc.) and informal offline credit (referring to loans obtained by farmers through friends and relatives, private moneylenders and underground moneylenders, etc.).

Despite the many advantages of digital credit mentioned above, digital credit is still subject to many risks because the Chinese digital credit market has not been established for a long time and is not well developed, especially in rural areas [13, 14]. First, rural areas are subject to many constraints in terms of the economic base and natural environment. The related policies can easily be poorly implemented, which has led many commercial digital credit financial institutions to voluntarily withdraw from the rural financial market. The lack of financial institutions at the rural grassroots level in terms of both number and scale, and the difficulty of their economic base to fit the financing needs of the relevant farmers, also essentially hinder the overall development of the rural economy. In contrast, the backward real economy will bring reverse stimulus to the development of rural digital credit, putting rural finance in a vicious circle situation [15, 16]. The risks faced by digital credit financial services for the rural economy are mainly in two aspects: on the one hand, digital credit provides financial support for rural economic development, and the number of funds granted needs to be systematically understood and objectively and scientifically measured for the demanders of funds, while the degree of rural financial development in China is relatively low, the nature of assets of rural residents is usually unique, farmers lack collateral for funds lending, and relevant credit records are There is a lack of good credit records. Therefore, the capital providers based on the Internet financial platform cannot conduct on-site inspections of rural residents. The information asymmetry between the two sides of the transaction is prominent, bringing specific challenges to credit risk prevention. Coupled with the weak credit awareness of rural residents and the lack of use of credit products, it is difficult for Internet financial platforms to scientifically measure credit-granting customers through big data. In addition, agricultural economic projects are different from ordinary projects in that agricultural products are easily disturbed by natural factors such as weather and have long recovery cycles. Traditional agricultural financial projects still risk higher overdue and inadequate debt rates, let alone digital credit finance, which relies only on credit loans and has more uncertainties [17]. On the other hand, the financial and operational risks of the digital credit platforms themselves should also be considered. With the dramatic increase of Internet finance platforms year by year, platform problems have also been intensified. Although there are no statistics related to problematic digital credit platforms in rural areas specifically, in terms of the digital credit industry as a whole, the number of challenging platforms has been increasing year by year. In addition, the amount of relevant loans involved has been growing, and agro-related finance platforms, as part of them, possess more uncertainties and more risks than other platforms. For example, in 2013, the cumulative number of problematic platforms was only 94, involving an inappropriate loan amount of 1.62 billion yuan; as of 2019, the number of challenging platforms has reached 4,130, involving a problematic loan amount of 33.24 billion yuan, which is 20 times more than in 2013. Therefore, the risk control of digital credit finance, especially the risk control of agricultural-related digital credit finance platforms, has become imminent [18, 19].

At the same time, rural e-commerce, as a representative application of information technology in rural areas, makes it possible for the digital dividend to better benefit rural areas. At the policy level, in recent years, the Chinese government has issued several documents and policies supporting rural e-commerce development, laying the foundation for rural e-commerce. The "2020 Research Report on Villages Engaged in E-Commerce Operations in China" released by Ali Research Institute, China’s largest Internet e-commerce platform, shows that as of September 2020, the number of villages engaged in e-commerce operations in China reached 5,425, accounting for 1% of the total number of administrative villages nationwide, mainly in provinces such as Zhejiang, Guangdong, and Jiangsu. The annual transaction value of towns engaged in e-commerce operations nationwide exceeded 1 trillion yuan, of which The annual transaction value of towns engaged in e-commerce business exceeds 1 trillion yuan, of which the annual transaction value of towns engaged in e-commerce business exceeds 100 million yuan reaches 745. The rapid rise of rural e-commerce is essentially the penetration of information technology and digital dividends into the rural market sector. In response to the digital compensation of rural e-commerce development, existing studies show that e-commerce improves farmers’ price search ability and reduces transaction costs [20, 21]. Through digital empowerment, e-commerce can increase profitability and turnover and help farmers increase their income [22–24]. Fintech can use digital information such as sales data and payment records left by farmers on e-commerce platforms to transform into credit scores and create prediction models and risk control strategies through big data technology to effectively reduce information asymmetry and transaction costs in credit transactions, thereby improving the level of credit supply and the efficiency of financial services. [16] constructed the Peking University Digital Inclusive Finance Index shows that inclusive digital finance offers the possibility for economically backward regions to achieve financial inclusion catch-up and lays the foundation for the majority of low- and middle-income people and disadvantaged groups to access financial services with broader coverage and greater depth of use.

Therefore, fintech, as a new financial industry, has gradually penetrated the county’s rural financial market. The question studied in this paper is whether digital credit in fintech development is an opportunity or a challenge for traditional bank credit? What is the relationship between the two? Meanwhile, due to the urban-rural digital divide and the relatively low financial literacy of rural households [25], the proportion of rural households using digital credit is low [26, 27]. So, while farmers engaged in rural e-commerce operations are the first to leave their digital footprints on digital platforms, do they also positively impact their digital credit access? To address the above issues, this paper empirically investigates rural residents in the China Family Panel Studies (CFPS) database based on 246 sample areas with a total of 26,275 valid questionnaires, using survey data for four years from 2017 to 2020. Using a multivariate Probit model and propensity score matching method, the impact of rural e-commerce development on farmers’ digital credit behavior is examined at the micro-level, specifically the complementary or substitution relationship between farmers’ digital credit and traditional bank credit and the impact of participation in e-commerce on the scale of farmers’ digital credit. The results are shown to be plausible after using robustness such as constructing instrumental variables and replacing models. The empirical results show that farmers who participated in e-commerce increased their access to digital credit size and total credit size by $0.922 million and $37.49 million, respectively, relative to those who did not participate in e-commerce.

We found that the popularity and development of Internet technology have promoted the rapid emergence of e-commerce in rural areas, which in turn has significantly changed the traditional financial service model of farmers and had a profound impact on the financial market in rural areas [28], and successively promoted the development of a series of financial innovations such as digital finance, mobile payment, and financial technology [29]. First, most of the micro studies on digital finance in rural areas have focused on online lending [15]. For example, Alipay users can use their "chanting" account for a certain amount of overdraft, and third-party payment platforms can also provide "microfinance" and "ant lending" with the credit points accumulated by users in the process of using mobile payments. The third-party payment platform can also provide small loans such as "Microfinance" and "Ant Borrowing" with the credit points accumulated by users in mobile payment. Thus, one of the marginal contributions of this paper is to examine the possible impact of rural e-commerce behavior on farmers’ lending while focusing on informal lending such as farmers’ digital credit. Second, can farmers’ e-commerce behavior improve farmers’ access to credit? What is the corresponding mechanism of action? The existing studies neither give enough attention nor provide clear answers to this question, and there is a lack of micro-level studies. As rural microeconomic agents, farmers can change their initial endowment through access to credit, maintain and expand their production scale, and thus alleviate poverty [30]. Therefore, when implementing rural revitalization strategies, it is essential to analyze whether farmers’ e-commerce behavior can enhance farmers’ credit access and its mechanisms at the micro-level. In addition, existing studies have not discussed the possible endogeneity of this issue, nor have they conducted a detailed analysis and empirical tests on the mechanism of farmers’ e-commerce behavior. This paper examines the impact of rural e-commerce development on rural households’ digital credit behavior at the micro-level using a multivariate Probit model and propensity score matching method with rural residents in the China Family Panel Studies (CFPS) database. Specifically, we examine the complementary or substitution relationship between rural digital credit and traditional bank credit and the impact of participation in e-commerce on the scale of rural digital credit. Thus, this paper has both theoretical and practical marginal contributions to the existing literature.

## II. Theoretical analysis and research hypothesis

### 1. The relationship between digital credit and traditional bank credit

It is worth noting that the potential challenges of digital credit to traditional bank credit should not be underestimated. First, although banks have an intrinsic motivation to change under the pressure of fintech, they are constrained by their shortcomings, such as path dependence, demand constraints, and mismatch between profit growth and growth needs, and are unable to achieve essential technological enhancements [31, 32]. Coupled with the lack of mastery of credit data, inefficient credit operations, complex procedures, and lack of Internet thinking, traditional banks lack the lasting motivation for intrinsic digital reform. In contrast, fintech-focused digital credit makes full use of information advantages to streamline credit processes, which puts more significant pressure on traditional banks’ credit operations. Studies have shown that the development of fintech has touched the "institutional dividend" and "price dividend" of conventional banks, intensified banks’ risk-taking, increased their risk appetite, weakened their profitability, and increased the risk of bankruptcy [33–36]. Second, fintech, as a disruptive and disruptive technological innovation, is bound to have a significant impact on traditional commercial banks [3, 37]. [38] argue that the combination of information technology and finance will significantly impact banks’ financial services and may change the shape of financial intermediation. Some studies even suggest that fintech, as a disruptive innovation, is bound to replace the traditional economic model in the long run [39]. Under the conventional bank lending model, information asymmetry leads to credit rationing as the norm, with high-quality but unsecured borrowers being rationed out of the credit market. Still, lending on fintech-based internet platforms, with the use of "soft information constraints" and "virtual collateral, However, lending on fintech-based internet platforms will push high-risk borrowers out of the market due to the use of "soft information constraints" and "virtual collateral." Digital credit is characterized by converting the applicant’s digital footprint, such as mobile payment and consumption information, into data in a concise period, creating predictive models and developing risk control strategies through big data technology. With the risk control strategy of digital finance, there is no need for loan applicants to apply at branches and provide asset collateral, which significantly reduces information asymmetry, transaction costs, and credit risks [40], thus mitigating transaction cost rationing and risk rationing in rural financial markets. Finally, digital credit can widen the boundaries of existing credit supply and enhance customer stickiness. Since digital credit applications are approved quickly and without collateral, it has the advantage of low transaction costs.

In contrast, traditional bank credit is time-consuming and has high transaction costs, and therefore has a high potential to be replaced by digital credit. [41] used Lending Club data to study the relationship between P2P and bank credit and found that P2P platforms borrow from the same groups banks choose to serve. Therefore, analyzing from the perspectives of banks’ limitations and the disruptive technological innovations of fintech, this paper argues that digital credit can substitute for traditional bank credit in the rural financial market.

Based on the above analysis, Hypothesis 1 is proposed in this paper.

**Hypothesis 1**: **There is a substitution relationship between digital credit and traditional bank credit**.

### 2. The relationship between farmers’ participation in e-commerce and their access to digital credit

Compared with farmers who do not participate in e-commerce, farmers who participate in e-commerce can not only expand their sales space and business scale to achieve increased income but also can more easily complete credit accumulation through the digital footprint left in the e-commerce platform, which in turn effectively alleviates the information asymmetry in financial transactions and reduces credit transaction costs, forming the advantage of obtaining loans in the Internet platform and making it easier to get digital credit.

Participation in e-commerce can bring important information and cost advantages because, firstly, e-commerce platforms have a large amount of merchant transaction information. Through a robust big data platform, they form more transparent and credible information, which can build a credit network system and provide a new channel for screening farmers’ credit information. Second, each loan under the traditional financing method requires dedicated personnel to investigate and grant credit, pre-credit review, and post-credit supervision, while the cost of pre-research and development, daily operation, and regular inspection of the e-commerce platform are fixed costs that do not change significantly with the number of people using the e-commerce platform, and its cost advantage is obvious. Third, Internet financing platforms that rely on financial technology can realize the tracking of lending to financing targets, effectively reducing search costs and helping to improve the targeting of credit services [42, 43]. [44] argue that the use of Internet platforms by financial institutions to collect information about farmers can significantly reduce information search costs, improve the reach of financial services in rural areas, and contribute to the improvement of creditThe participation of farmers in e-commerce is conducive to the formation of a digital footprint. Financial technology companies supported by e-commerce platforms can rely on the large amount of merchant transaction information stored on the platforms to convert it into credit scores, which can prompt farmers who initially lacked credit to complete credit accumulation, and complete information review, line matching and loan issuance between the two parties in a concise time with the help of information technology such as big data, cloud computing, and artificial intelligence. To achieve the goal of using the e-commerce platform as a medium for both the demand side and the supply side of funds. This transaction process effectively alleviates the information asymmetry in traditional credit transactions, reduces the transaction costs of lending, and helps farmers gain access to digital credit opportunities.

In addition, income is an essential factor in access to credit [45]. It has been shown that farmers’ participation in e-commerce has a significant income-increasing effect [46], promoting more digital credit support for farmers through the income effect. From the demand side, farmers’ participation in e-commerce expands their sales channels and business scale. The introduction of e-commerce has significantly changed farmers’ traditional operating expenditure structure, who need to increase physical capital such as product packaging, express distribution, advertising and marketing, and platform usage fees, which increases the liquidity capital constraint and invariably makes farmers’ credit demand stronger. From the supply side, the higher the household income, the higher its performance ability and credit level, the easier it is to obtain larger loans [47]. Income is one of the essential criteria financial institutions use to screen potential borrowing farmers due to information asymmetry between borrowers and lenders before lending. A high income level implies a high repayment rate, and it becomes a rational choice for financial institutions to exclude farmers with low-income levels to avoid credit risk. The introduction of rural e-commerce has increased the sales path of farmers’ agricultural products and contributed to the growth of e-commerce sales while changing the unfavorable situation of farmers who used to be price takers [48], creating an income growth effect. In addition, the development of rural e-commerce is typically aggregative and can generate more significant supply-side economies of scale [49, 50], resulting in more robust sustainability of farmers’ income growth and better access to digital credit support for farmers.

Based on the above analysis, this paper proposes research hypothesis 2.

**Hypothesis 2**: **Farmers’ participation in e-commerce has a positive impact on access to digital credit**.

## III. Data analysis and model construction

### 1. Data source

The data used in this paper are from the China Family Panel Studies (CFPS) 2017–2020 quadrennial database. China Family Panel Studies (CFPS) aims to reflect social, economic, demographic, educational, and health changes in China by tracking and collecting data at three levels: individual, household, and community, and to provide a database for CFPS focus on the economic and non-economic well-being of Chinese residents, as well as many research topics including economic activities, educational outcomes, family relationships, and family dynamics, population migration, and health, etc. It is a nationwide, large-scale, multidisciplinary social tracking survey project. There are four main types of CFPS questionnaires: community questionnaire, household questionnaire, adult questionnaire, and child questionnaire, and on this basis, various types of questionnaires such as long questionnaire, a short questionnaire, proxy questionnaire, and telephone questionnaire have been developed for different types of household members. Therefore, this paper selects data from CFPS 2017–2020 to study the impact of rural e-commerce development on farmers’ digital credit behavior. Specifically, the sample is limited to rural households. Then the data is matched to 28,235 households in the four periods of CFPS2017-2020, which is more authoritative and representative because of the newer data and wider coverage. Excluding incomplete and logically confusing questionnaires, 26725 valid questionnaires were obtained from 246 sample counties in 19 provinces, including Shanxi, Liaoning, Jiangsu, Anhui, Shandong, Henan, Guangdong, Hunan, Hubei, Sichuan, Yunnan, Gansu, Hebei, and Jilin. Among them, 37.8% (10,102) were e-commerce farmers and 62.2% (16,622) were non-e-commerce farmers, as shown in Table 1.

**Table 1:** Distribution of sample farmers.

Years	E-commerce farmers (households)	Digital Credit Ratio	Non-electric farmers (households)	Digital Credit Ratio	Total
2017	2541	21.22%	4175	11.52%	6716
2018	2515	20.25%	4122	12.49%	6637
2019	2503	22.55%	4155	10.18%	6658
2020	2543	23.00%	4170	11.41%	6713
Total	10102	-	16622	-	26725

### 2. Variable selection

#### i. Explained variable

Digital credit in this paper refers to loans from financial institutions obtained by farmers through Internet channels rather than offline channels, which include online loans obtained from e-commerce platforms and online loans from banks. It does not have offline credit from traditional banks (referring to farmers’ access to conventional offline loans from formal financial institutions, such as rural commercial banks, agricultural banks, and village banks) and informal offline credit (referring to farmers’ access to loans through friends and relatives, private moneylenders and underground moneylenders). In this paper, whether farmers receive credit or not is selected as the explanatory Variable. Farmers are considered to have digital credit behavior if they have obtained digital recognition and are assigned a value of 1, otherwise 0. Traditional bank credit behavior and informal credit behavior are defined in the same way. In studying the effect of farmers’ participation in e-commerce on the scale of digital credit acquisition, the explanatory variables are the scale of digital credit acquired by farmers’ households and the total scale of honor, and the full scale of credit is the sum of the credit earned by farmers through various channels.

#### ii. Core explanatory variables

The core explanatory variable of this paper is whether the farmers participate in rural e-commerce. The value of "yes" is defined as participating in rural e-commerce, assigned as 1. Otherwise, it is not participating in rural e-commerce, which is given as 0.

#### iii. Control variables

In studying the relationship between digital credit, traditional bank credit, and informal lending, the control variables selected in this paper include four aspects: demographic characteristics of farmers, production and business, social capital, and formal financial supply. The demographic characteristics chosen in this paper are the farmers’ age, education level, and household demographic burden rate. Since digital credit is an emerging financial supply model, theoretically, the younger and better-educated farmers are more likely to accept it. Farmers’ production and operation is an essential factor affecting their credit demand and access to credit support. It has been found that the higher the income level and the stronger the repayment ability, the higher the availability of credit for farmers. This paper chooses the area planted with flowers and trees and the total income of flower and tree businesses as the proxy variables of farmers’ production and business situation. Since social capital as an intangible asset plays a vital role in farmers’ credit access, the human relations, social network, social trust, and reputation embedded in social capital can significantly reduce the information asymmetry and transaction costs in the household credit market, so this paper selects credit reputation, social relations, and household human exchange expenditure to reflect farmers’ social capital characteristics. Finally, this paper uses the distance of farm households to the nearest financial institution to control for differences in formal financial supply. The closer the village is to the financial institution, the lower information asymmetry and legal credit transaction costs. The higher the likelihood that farmers will obtain credit.

This paper also uses the Propensity Score Matching (PSM) model to examine the effect of farmers’ participation in e-commerce on their access to credit size. At this time, the variables need to be re-matched. The matching process is designed to maximize the number of identifiable variables that could theoretically influence farmers’ participation in e-commerce and credit access and add, delete, and combine variables based on the matching results to fully utilize the sample and improve the matching effect. After several variable selections, the matching variables chosen in this paper are the gender of the household head, age, health, education level, whether relatives or friends are working in the government or banking sector, net income, and entrepreneurial experience.

### 3. Descriptive statistical analysis

The results of descriptive statistics of the main variables in this paper are shown in Table 2. It can be found that 17.2% of the sample farmers obtained digital credit through online channels. In comparison, the shares of loans obtained through traditional bank credit and informal credit methods were 36.2% and 6.0%, respectively. Regarding the scale of credit received by farmers, the mean value of digital credit was 0.752 million yuan, accounting for 12.7% of the total scale of household credit. In this paper, we test for group differences between whether farmers participate in e-commerce and the size of credit acquisition. The t-test in Table 2 shows that the digital credit size and total credit size obtained by e-commerce farmers are significantly higher than those of non-e-commerce farmers, with the average digital credit size of e-commerce farmers and non-e-commerce farmers being $12.25 million and $0.102 million, respectively, the former being $11.23 million higher and the total credit size $37.22 million higher than the latter, and this difference is significant at the 1% level. It is thus intuitively perceived that whether or not farmers are involved in e-commerce may be an essential reason for the difference in the size of credit access.

**Table 2:** Descriptive statistics and tests of variance.

Variable Name	Variable Definition	E-commerce Farmers		Non-electric Farmers		T-test
		Average value	Standard deviation	Average value	Standard deviation	
Access to digital credit or not	Access to credit using fintech = 1, no = 0	0.268	0.265	0.125	0.248	1.154***
Access to traditional bank credit	Obtaining credit from traditional banks = 1, No = 0	0.518	0.189	0.318	0.141	1.615**
Access to informal credit	Obtaining credit from informal channels = 1, No = 0	0.075	0.821	0.051	0.985	1.681**
Participation in e-commerce	Selling products through e-commerce platforms = 1, No = 0	0.780	0.515	0.656	0.553	2.618***
Size of digital credit	Scale of digital credit received by farmers (million yuan)	1.225	3.315	0.102	0.156	1.132***
Total credit size	The total size of credit received by farm households (million yuan)	8.561	14.156	4.156	9.013	3.165***
Age	Age of household head (years)	46.52	11.562	16.81	11.862	2.814***
Education level	Years of education of household head (years)	9.862	3.485	9.516	3.468	2.618***
Population burden rate	Number of non-labor force as a percentage of total household size	0.562	0.224	0.518	0.148	1.612***
Gender	Gender of household head, male = 1, female = 0	0.922	0.451	0.923	0.516	2.615***
Health level	Health = 1; frail and sickly = 2; suffering from serious diseases = 3	1.281	0.381	1.268	0.345	3.186***
Income from flowers and trees	Total income from flower and tree business (million yuan)	84.182	125.148	82.176	22.156	2.165***
Creditworthiness	Experience of repaying loans on time in the last two years = 1, no = 0	0.350	0.161	0.360	0.161	3.164***
Social relationship	Have social relationship with financial institutions or government = 1, no = 0	0.526	0.515	0.516	0.483	2.485***
Human relations expenses	Family expenses for human affairs (million yuan)	1.816	2.158	1.862	2.265	1.121**
Family and friends relationship	Have relatives or friends working in government or bank = 1, no = 0	0.635	0.566	0.615	0.122	0.532
Net income	Net income from flower and tree industry (10,000 yuan)	35.135	49.315	33.816	43.812	0.682
Financial institution distance	Distance of farm household from the nearest rural financial institution (km)	2.561	1.245	2.261	1.248	1.891**
Entrepreneurial experience	Household head has entrepreneurial experience = 1, no = 0	0.584	0.452	0.551	0.418	2.185***

Note

***

**

* denote 1%, 5%, and 10% significance levels, respectively.

In this paper, we test for group differences between whether farmers participate in e-commerce and the scale of credit acquisition. The t-test in Table 3 shows that the digital credit size and total credit size obtained by e-commerce farmers are significantly higher than those of non-e-commerce farmers, with the average digital credit size of e-commerce farmers and non-e-commerce farmers being $10.09 million and $0.96 million, respectively, with the former being $10.13 million higher than the latter and the total credit size being $35.55 million more elevated. This difference is significant at the 1% level. It is thus intuitively perceived that whether farmers participate in e-commerce may be an important reason for the difference in the size of credit access.

**Table 3:** Estimation results of the interrelationship between digital credit and traditional bank credit.

Explanatory variables	(1) Digital Credit		(2) Traditional bank credit		(3) Informal credit	
	Coefficient	Standard error	Coefficient	Standard error	Coefficient	Standard error
Participation in e-commerce	0.041***	0.042	0.030***	0.012	0.002*	0.322
Age	-0.023**	0.015	-0.029	0.026	-0.051	0.063
Education level	0.062**	0.548	0.089	0.056	0.025	0.029
Population burden rate	0.361	0.518	-0.248	0.382	0.419**	0.698
Creditworthiness	0.007***	0.424	0.053***	0.226	0.056*	0.226
Social relationship	0.651	0.851	0.109	0.312	-0.260	0.326
Human relations expenses	0.300**	0.122	0.040	0.037	0.062***	0.019
Financial institution distance	0.035***	0.072	0.007	0.052	0.152	0.0256
Constant term	-0.322	0.826	-0.056*	0.526	-0.107***	1.015
Error term correlation coefficient		*Ρ*12			-0.426***(0.126)	
		*ρ*13	0.553***(0.271)			
		*ρ*23	0.326***(0.178)			
The maximum likelihood function value			-374.974			
Wald chi^2^ (30)			105.41***			
LR test			chi^2^ (3) = 25.136; Prob > chi^2^ = 0.000			
Observations	16225					

Note

***

**

* denote 1%, 5%, and 10% significance levels, respectively; standard errors are in parentheses; ρ_ij_(i,j = 1,2,3) represents the correlation coefficient between the error term ε_i1_、ε_i2_、ε_i3_ of equation (i) and equation (j); if the coefficient is positive and significant, it indicates a complementary relationship between the two credit behaviors of farmers; if the coefficient is negative and powerful, it suggests a substitution relationship between the two credit behaviors of farmers.

### 4. Model construction

i. In this paper, we refer to [51] and develop a joint cubic equation model to classify the credit behavior of farmers into three types of credit: digital credit, traditional bank credit, and informal credit, and estimate the influence relationship between the three with the help of a ternary Probit joint cubic equation model, using the maximum likelihood function method (Simulated Maximum Likelihood [52] was used to solve the model. This paper focuses on the relationship between digital credit and traditional bank credit. If the relationship between digital credit and traditional bank credit is an alternative, the error term correlation coefficient should be negative. If the relationship between digital credit and traditional bank credit is complementary, the error term correlation coefficient should be positive. The specific econometric model is as follows.


$$y_{1i}^{*}=\beta_{1}^{\prime }X_{1i}+\varepsilon_{1i}$$ (1)

$$y_{2i}^{*}=\beta_{2}^{\prime }X_{2i}+\varepsilon_{2i}$$ (2)

$$y_{3i}^{*}=\beta_{3}^{\prime }X_{3i}+\varepsilon_{3i}$$ (3)

$$y_{1i}=\{\begin{array}{ll} 1 & y_{1i}^{*}\geq 0\\ 0 & y_{1i}^{*}<0 \end{array}$$ (4)

$$y_{2i}=\{\begin{array}{ll} 1 & y_{2i}^{*}\geq 0\\ 0 & y_{2i}^{*}<0 \end{array}$$ (5)

$$y_{3i}=\{\begin{array}{ll} 1 & y_{3i}^{*}\geq 0\\ 0 & y_{3i}^{*}<0 \end{array}$$ (6)

$y_{1i}^{*}$ represents the hidden Variable of digital credit behavior of sample farmers, *y*_1*i*_ represents the decision variable of whether farmers participate in digital credit or not. $y_{2i}^{*}$ Represents the hidden variables of farmers’ traditional bank credit behavior, *y*_2*i*_ represents the decision variables of whether farmers participate in traditional bank credit. $y_{3i}^{*}$ Represents the hidden Variable of farmers’ participation in informal lending behavior and *y*_3*i*_ represents the decision variable of whether farmers participate in informal credit.

ii. Propensity score matching model. For the effect of farmers’ participation in e-commerce on the scale of credit acquisition, this paper divides the study population into an experimental group (farmers participating in e-commerce) and a control group (farmers not participating in e-commerce). It uses Propensity Score Matching (PSM) to assess the effect of credit acquisition after farmers participate in e-commerce. The experimental and control group samples were matched according to the matching principle. The differences between the two groups were controlled to the maximum extent possible so that the matching characteristics of the two groups of farmers were as similar as possible. The control group simulated the counterfactual state of the experimental group; that is, the credit size obtained by farmers who did not participate in e-commerce was replaced by the credit size received by farmers who did not participate in e-commerce, thus getting a "clean "farmer credit size differences, i.e., estimating the Average Treatment Effect of the Treated (ATT). The specific model is as follows.


$$Y_{i}=\alpha +\delta D_{i}+\beta X_{i}+\varepsilon_{i}$$ (7)

In Eq (7) *Y*_*i*_ denotes the total size of credit received by farmers. *D*_*i*_ Indicates whether farmers are involved in e-commerce and *D*_*i*_ = 1 if yes, and *D*_*i*_ = 0 if otherwise. *X*_*i*_ is the other explanatory variables, *α* is the constant term, and *ε*_*i*_ is the random disturbance term. The enhancement effect of credit size after farmers’ participation in e-commerce can be measured when they are randomly assigned to the experimental and control groups. For the e-commerce farmers, the average treatment effect (ATT) of their credit size is:

$$ATT=E(Y_{1}|D=1)-E(Y_{0}|D=1)=E(Y_{1}-Y_{0}|D=1)$$ (8)

Eq (8) *Y*_1_ denotes the credit size obtained by farmers after participating in e-commerce and *Y*_0_ represents the credit size obtained by farmers without participating in e-commerce. Thus, ATT indicates the net effect of the credit size received by farmers after participating in e-commerce. To increase the robustness of the assessed impact, the standard error of ATT is inferred using self-sampling (Bootstrap) 500 times.

iii. instrumental variables approach with Tobit model since there may be factors in the random error term that affect both farmers’ digital credit behavior and farmers’ traditional bank credit behavior, which may lead to biased estimation results. Considering the possible endogeneity problem caused by omitted variables, the instrumental variables are chosen to try to solve the potential endogeneity problem, which must be at least positively correlated with farmers’ participation in e-commerce behavior and not significantly correlated with farmers’ access to digital credit support, i.e., consistent with homogeneity. Specifically, the instrumental Variable is based on the prefectural and municipal mayors database in the People’s Republic of China (2017–2020), referring to[53]. However, since e-commerce is only classified as economic work in the job arrangement of mayors, whether the head of the prefecture-level administrative region (mayor) has an education background in economics and whether they have work experience in e-commerce are selected as instrumental variables for farmers’ participation in e-commerce behavior. After completing some of the missing data, a two-step IV-Probit model is applied for estimation. After that, this paper considers that many households do not start their own business, so whether they engage in e-commerce activities satisfies the left-hand side truncation feature. Therefore, we continue to re-estimate the data using the Tobit model in the endogeneity testing stage to verify the robustness of the results.

## IV. Empirical results and discussion

### 1. Examination of the relationship between digital credit and traditional bank credit

Table 3 below shows the results of the baseline regressions estimated using the ternary Probit model. For the reader’s understanding, the results presented in the following tables, including Table 3 below, are the results of estimating the marginal effect. The first column of the explanatory Variable is digital credit, the second column is traditional bank credit, and the third column is informal credit. It can be seen that the coefficients of participation in e-commerce are 0.041, 0030, and 0.002 and significant at the 1% level, respectively, and the model LR test rejects the original hypothesis, showing that the model is valid overall. The conclusion tentatively suggests that farmers’ participation in e-commerce behavior can significantly increase the likelihood of farmers’ access to digital credit support. In addition, most of the remaining control variables are significant at a 1% level of significance, such as education level and credit reputation, which can enhance the possibility of farmers’ access to digital credit. In contrast, farmers’ age shows a significant negative relationship with digital credit access.

In Table 3 *ρ*_12_,*ρ*_13_,*ρ*_23_ is the correlation coefficient between the error terms of the equation, and all of them reject the original hypothesis that digital credit to farmers, traditional bank credit, and informal credit are independent of each other at 1% significance level, indicating that the three are interacting with each other. From the likelihood ratio test results, the original hypothesis that the correlation coefficients of the error terms are simultaneously zero is rejected at the 1% significance level, indicating that it is reasonable to choose the ternary model for estimation. The results in Table 3 show that the estimated value is negative and significant at the 1% level, indicating a substitution relationship between digital credit and traditional bank credit behavior of farmers, thus verifying the hypothesis of this paper.1 This means that although there is a theoretical possibility of substitution or complementarity between digital credit and traditional bank credit, the estimated results of this paper show that there is a significant substitution relationship between the two. Further, it can be seen that the estimate *ρ*_13_、*ρ*_23_ is positive and practical at the 1% level, indicating that there is a complementary relationship between digital credit and informal credit and relaxed credit and traditional bank credit.

Further, it can be found that e-commerce participation contributes to farmers’ access to digital credit and is significant at the 1% level. Furthermore, the age and education level of the household head has a considerable effect on the access to digital credit by farm households, but not on the traditional bank credit behavior and informal credit behavior. This implies that in the era of financial technology, the age and education level of the household head are essential factors influencing farmers’ choice of digital credit, and farmers who are younger and better educated are more receptive to new things such as the Internet and e-commerce, have a relatively more minor digital divide, and are more likely to adopt digital credit. In addition, credit reputation significantly and positively affects farmers’ digital credit acquisition, possibly because farmers’ credit history is an essential criterion for fintech platforms to screen their customers, and farmers with a good credit history are more likely to obtain digital credit from fintech platforms.

### 2. Instrumental variables and endogeneity analysis

Although the above results suggest that rural e-commerce development and farmers’ digital credit behavior are correlated, there may also be endogeneity between the two. Endogeneity may arise from several sources: first, there may be a reverse causality mechanism, where e-commerce has an impact on residents’ entrepreneurial choices, leading to greater participation in digital credit behavior, but residents’ entrepreneurial behavior may also lead to the development of local e-commerce, although the extent to which micro-entities influence the indicators at the regional level may be limited. Second, there may be a problem with omitted variables. For example, there is no multi-year data to exclude invisible characteristics of individuals and households, which may omit variables related to farmers’ digital credit behavior, such as personal characteristics and local institutions. These omitted variables may be associated with the explanatory Variable of farmers’ e-commerce development level, thus causing endogeneity problems.

In this paper, on the one hand, as many controllable variables as possible are included in the baseline regressions to reduce the effects of omitted variables, such as GDP per capita to control for local economic development, fiscal expenditure share to head for the level of local marketization, and industry and occupation dummy variables to control for other unobservable characteristics of different types of individuals; on the other hand, instrumental variables are selected to try to address possible endogeneity issues. The instrumental variables must meet at least a positive correlation with the participation of farmers in e-commerce behavior and no significant correlation with the access of farmers to digital credit support, i.e., they are consistent with homogeneity. Referring to [54, 55], this paper argues that whether the head of a prefecture-level municipality (mayor) has a background in economics education and whether they have work experience in e-commerce can serve as an instrumental variable for whether farmers engage in e-commerce behavior. The reasons for this are as follows.

The mayor’s attention as a "handful" has a vital role in promoting e-commerce development and positively correlates with farmers’ participation in e-commerce. The mayor is in charge of the city’s economy and can control finances, personnel appointments, and dismissals. Although China’s social governance model is "the rule of law" rather than "rule of man," the mindset and influence of leaders cannot be ignored. In the case of e-commerce development, whether the mayor has previous e-commerce and "Internet+" thinking may directly impact the growth of e-commerce in the city [56], using American cities as an example suggests that the attention of a city’s top executive has a catalytic effect on e-commerce development. And what factors determine this governance mindset and approach of mayors? This paper argues that if mayors had had e-commerce related educational backgrounds and work experiences before they took office, then they will have a deeper understanding of developing e-commerce, which will shape their mindset of focusing on e-commerce and expanding e-commerce, indicating that the more the chief executive of the area is concerned about e-commerce development, the higher the probability of farmers participating in e-commerce, which is in line with the correlation hypothesis of Ⅳ.

On the other hand, the mayor’s e-commerce related educational experience and work experience positively correlate with whether rural residents engage in e-commerce behavior but not with the availability of digital credit support for farm households. Therefore, the e-commerce-related educational and work experiences of mayors at the region’s overall level do not directly impact whether individual farm households receive digital credit support consistent with the homogeneity hypothesis of IV. Therefore, this paper uses whether or not the head of a prefecture-level municipality (mayor) has an educational background in economics and whether or not they have work experience in e-commerce as instrumental variables for whether or not farmers engage in e-commerce behavior.

Specifically, the instrumental variables were established based on the database of prefectural and municipal mayors in the People’s Republic of China (2017–2020) concerning the study of [53]. However, since e-commerce is only classified as an economic job in the mayor’s work schedule, whether the head of the prefecture-level administrative region (mayor) has an educational background in economics and has work experience in e-commerce was selected to serve as an instrumental variable for farmers’ participation in e-commerce behavior. The results of the two-step IV-Probit model are presented in Table 4, where the first stage F-values are 98.7, 92.6, and 94.9, which are much higher than the practical value of 10, indicating that the hypothesis of weak instrumental variables is rejected. Furthermore, the Wald test shows that the original idea is denied in all regression results, indicating that using instrumental variables is significantly different from not using instrumental variables. However, the effect of e-commerce on farmers’ digital credit behavior is still considerably positive, except that the estimated coefficients and standard errors have changed. The sign and significance level of the core parameters remains consistent with the baseline regression results.

**Table 4:** Model estimation results after using instrumental variables.

Variables	Phase I	Phase II	Phase I	Phase II	Phase I	Phase II
	Participation in e-commerce	Digital Credit	Participation in e-commerce	Traditional bank credit	Participation in e-commerce	Informal credit
Participation in e-commerce		0.074***		0.024***		0.064*
		(0.026)		(0.254)		(0.256)
Age	-0.015***	-0.012**	-0.063**	-0.048**	-0.045	-0.071
	(0.234)	(0.125)	(0.015)	(0.148)	(0.154)	(0.231)
Education level	0.758***	0.848***	0.042**	0.048*	0.0156	0.0152
	(0.267)	(0.185)	(0.068)	(0.034)	(0.152)	(0.322)
Population burden rate	0.058*	0.045	-0.002*	-0.074	0.548**	0.691**
	(0.267)	(0.234)	(0.267)	(0.216)	(0.317)	(0.253)
Creditworthiness	0.328**	0.372***	0.715**	0.814**	0.002**	0.002*
	(0.265)	(0.238)	(0.218)	(0.216)	(0.157)	(0.225)
Social relationship	-0.056*	-0.074	0.164	0.174	-0.521*	-0.578
	(0.315)	(0.168)	(0.140)	(0.165)	(0.261)	(0.256)
F-value	98.7		92.6		94.9	
	*ρ*12		-0.574***(0.01)			
Error term correlation coefficient	*ρ*13		0.31***(0.001)			
	*ρ*23		0.374**(-0.171)			
Maximum likelihood function value	-348.74					
Wald chi2 (30)	375.41***					
LR Inspection	chi2 (3) = 58.145; Prob > chi2 = 0.000					

Note

***

**

* denote 1%, 5%, and 10% significance levels, respectively; standard errors are in parentheses.

Since many households do not start a business, whether or not a family engages in e-commerce activities satisfies the left-hand side truncation feature. Therefore, the Tobit model is used for estimation, and the control variables are consistent with Tables 3 and 5 reports the estimation results in the Tobit model. It can be seen that the effect of e-commerce level on farmers’ digital credit behavior is still significantly positive, except for changes in the estimated coefficients and standard errors, the signs and significance levels of the core parameters remain consistent with the baseline regression results, which implies that the substitution relationship between farmers’ digital credit and traditional bank credit still holds after changing the model, further illustrates that the above estimation results are robust.

**Table 5:** Robustness test: Tobit model regression results.

Explanatory variables	(1) Digital Credit		(2) Traditional bank credit		(3) Informal credit	
	Coefficient	Standard error	Coefficient	Standard error	Coefficient	Standard error
Participation in e-commerce	0.048***	0.048	0.018***	0.048	0.018*	0.315
Age	-0.014**	0.048	-0.015**	0.077	-0.040	0.018
Education level	0.874***	0.502	0.075*	0.0052	0.014	0.005
Population burden rate	0.084	0.348	-0.015	0.484	0.646**	0.815
Creditworthiness	0.456***	0.149	0.848**	0.518	0.048*	0.448
Social relationship	-0.013	0.041	0.018	0.548	-0.047	0.405
Human relations expenses	0.048***	0.074	0.074	0.002	0.045	0.104
Financial institution distance	0.078	0.315	0.040***	0.063	0.104	0.005
Constant term	-0.241	0.576	-1.071	0.810	-2.871***	1.015
Adj.R^2^	0.752		0.761		0.855	
LR test			chi^2^ (3) = 28.512; Prob > chi^2^ = 0.000			

Note

***

**

* denote 1%, 5%, and 10% significance levels, respectively; standard errors are in parentheses.

### 3. Impact of participation in e-commerce on digital credit and total credit size for farmers

#### i. Related index matching quality detection

Considering the matching effect and sample usage, five matching methods were selected in this paper: nearest neighbor matching (1–5 matching), near-neighbor matching (1–10 matching), radius matching, kernel matching (with a bandwidth of 0.06), and local linear regression matching. As can be seen from Table 6, the standard deviations of the mean values of the variables decreased significantly after matching, and all standard deviations were within 10%. The t-test indicated no significant differences between the experimental and control farmers on most variables after matching, and the changes in p-values also showed that the control variables were well balanced.

**Table 6:** The standard deviation of the mean values of variables before and after matching.

Variable	Before and after matching	Mean value of experimental group	Control group mean value	Standard deviation	Standard deviation reduction	T	P>|T|
Gender	Before Matching	0.874	0.625	19.52	86.2	1.61	0.0702
						-0.22	0.645
	After Match	0.822	0.745	-5.5			
Age	Before Matching	37.864	44.718	-60.178	95.1	-5.26	0.000
	After Match	36.448	38.151	-5.748		-0.46	0.268
Health level	Before Matching	1.152	1.153	-16	115	-1.15	0.265
	After Match	1.123	1.1263	0		0.00	1.000
Education level	Before Matching	9.352	8.526	28.5	71.1	2.748	0.026
	After Match	9.145	9.852	9		0.263	0.448
Entrepreneurial experience	Before Matching	0.552	0.322	-12.14	89.1	3.265	0.001
						-0.52	0.150
	After Match	0.570	0.263	0.6			
Flower and tree planting area	Before Matching	11.235	17.526	-15.6	72.1	-1.745	0.115
	After Match	11.041	10.023	3.5		0.718	0.714
Relatives and friends	Before Matching	0.515	0.315	30.5	35.4	2.156	0.052
						-1.152	0.217
	After Match	0.482	0.574	-19.1			
Net income	Before Matching	39.784	27.745	17.5	41.7	1.15	0.115
	After Match	35.263	40.125	-9.5		-0.152	0.411

As shown from Table 7, taking nearest neighbor matching (1 to 5 matching) as an example, PseudoR2 decreased from 0.121 before matching to 0.005 after comparing, LR statistic decreased from 49.22 before 3.49 after checking. Furthermore, mean Bias (Mean Bias) fell from 29.5 before corresponding to 5.0. Median Bias (Med Bias) decreased from The matching significantly reduced the difference between the control and experimental groups and minimized the sample selection bias, indicating that the control and experimental groups had had a good matching effect.

**Table 7:** Sample matching methods and their balance test results.

Matching method	Pseudo R^2^	LR statistic	Mean Bias	Med Bias
Before matching	0.121	49.22	29.5	27.5
Nearest Neighbor Matching (1 to 5 matches)	0.005	3.49	5.0	4.1
Nearest Neighbor Matching (1 to 10 matches)	0.006	3.15	4.5	3.5
Radius matching	0.015	6.15	6.2	6.1
Kernel matching (bandwidth of 0.06)	0.002	1.98	3.7	3.5
Local linear regression matching	0.015	12.74	8.2	8.2

#### ii. Effect estimates of the impact of farmers’ participation in e-commerce on digital credit and the total size of access to credit

Based on the matched successful sample, this paper further estimates the credit size enhancement effect of farmers’ participation in e-commerce. Table 8 shows the digital credit scale, total credit scale and average processing effect (ATT) of the experimental group and the control group under the five matching methods. It can be seen that the results obtained by various matching methods are very close. After farmers participate in e-commerce, the scale improvement effect of digital credit is very obvious, which is significant at the level of 1%. At the same time, the effect of increasing the credit scale obtained by farmers after participating in e-commerce is also obvious, which is significant at the level of 5%. From the average value of the calculation results of the above five matching schemes, if e-commerce farmers do not participate in e-commerce, their digital credit scale and total credit scale are 18200 yuan and 458600 yuan respectively. If you participate in e-commerce, the scale of digital credit and the total scale of credit will increase to 12600 yuan and 858300 yuan respectively. The net effects of participating in e-commerce to obtain digital credit and the total scale of credit are 92200 yuan and 374900 yuan respectively. This result reflects that with the continuous popularization of the Internet, rural e-commerce based on "villages engaged in e-commerce" has developed rapidly, so that farmers can not only enjoy digital dividends, but also obtain financial benefits with increased credit scale. The application of financial technology has further expanded the coverage and availability of rural financial services, Thus, it effectively alleviates the credit constraints faced by farmers, and hypothesis 2 of this paper is verified.

**Table 8:** Estimated results of the impact of participation in e-commerce on the credit size of farm households.

Matching method	Digital credit size (million yuan)			Total credit size (million yuan)		
	Experimental group	Control group	ATT	Experimental group	Control group	ATT
Nearest Neighbor Matching (1 to 5 matches)	1.145	0.1526	0.256***	8.015	4.714	3.826**
Nearest Neighbor Matching (1 to 10 matches)	1.156	0.265	0.718***	8.157	4.185	3.256**
Radius matching	1.0123	0.171	0.841***	8.041	4.178	3.718**
Kernel matching (bandwidth of 0.06)	1.151	0.718	0.718***	8.718	4.105	3.748**
Local linear regression matching	1.152	0.1856	0.526***	8.124	4.186	3.124**
Mean value	1.715	0.1182	0.715	8.177	4.748	3.715

Note: Estimation results of ATT values were obtained by the self-help method with 500 replicate samples.

***

**

* denote 1%, 5%, and 10% significance levels, respectively.

#### iii. Differences in the size of access to credit for e-commerce farmers with different endowment characteristics

For further analysis, are there differences in the size of credit received by e-commerce farmers? This paper examines heterogeneity in terms of differences in the endowments of participating e-commerce farmers. In this paper, capital endowments are divided into physical capital, human capital, and social capital. Physical capital uses operating expenditure and flower and tree planting area as proxy variables, while human capital and social capital use education level and human expenditure as proxy variables, respectively. Restricted by the sample size, this paper is divided into two subsamples for each grouping variable for all e-commerce farmers’ samples to obtain a better matching effect. Business expenditure, flower and tree planting area, and human interaction expenditure were split into two groups of "greater than the mean" and "less than the mean" according to the mean level of the whole sample, and education level was answered as "elementary school and below" and "junior high school." The two options of "primary school and below" and "junior high school" were unified as junior high school and below. On this basis, propensity score matching was estimated for the above-grouped variables.

The estimation results in Table 9 show that the digital credit size and total credit size received by farmers with operating expenditure levels above the mean increased by $0.82 million and $66.75 million, respectively after they participate in e-commerce. In comparison, those below the standard only increased by $0.525 million and $52.22 million, respectively. Farmers with flower and tree cultivation area above the mean increased their access to digital credit scale and total credit scale by 0.841 million yuan and 39.49 million yuan, respectively, while farmers below par increased their digital credit scale and full credit scale by only 0.552 million yuan and 27.82 million yuan respectively, i.e., the richer the physical capital, the larger the credit scale obtained by the e-commerce farmers. The digital credit size and total credit size received by farmers with high human capital endowment increased by $0.925 million and $75.22 million, respectively, after participating in e-commerce, while the digital credit size and total credit size obtained by e-commerce farmers with low human capital endowment increased by only $0.461 million and $64.5 million, respectively, after participating in e-commerce, indicating that the enhancement effect of participation in e-commerce on credit size varied according to human capital endowment goes. In addition, the digital credit size and total credit size of e-commerce farmers rich in social capital increased by $0.08 million and $67.55 million, respectively, while those poor in social capital increased by only $0.462 million and $45.51 million, respectively, i.e., the richer the social capital of e-commerce farmers, the larger the size of credit they received. Social capital is the concentration of contacts, networks, and exchangeable resources; the higher the level of social capital, the more conducive to farmers to expand customer resources and enhance the sales income of flowers and trees. The e-commerce platform can convert farmers’ sales and income information into credit scores, which improves farmers’ credit levels and helps to enhance the scale of digital credit access for farmers with high social capital levels.

**Table 9:** Differences in the size of access to credit for e-commerce farmers with different endowment characteristics.

Grouping variables		Digital credit size (million yuan)			Total credit size (million yuan)		
		Experimental group	Control group	ATT	Experimental group	Control group	ATT
Physical capital: level of expenditure	Larger than average	0.886	0.098	0.822***	12.882	6.118	6.675***
	Less than the mean	0.654	0.073	0.525**	11.212	6.044	5.222***
	Difference	0.232	0.025	0.257	1.671	0.074	1.597
Physical capital: Flower Tree planting area	Larger than average	0.955	0.133	0.841***	11.828	7.935	3.949**
	Less than the mean	0.619	0.078	0.552**	10.276	7.500	2.782**
	Difference	0.336	0.055	0.281	1.551	0.435	1.117
Human Capital	Junior high school and above	1.065	0.094	0.925***	13.177	5.603	7.522***
	Junior high school and below	0.575	0.091	0.461**	10.540	4.077	6.450***
	Difference	0.490	0.003	0.486	2.637	1.526	1.111
Social Capital	Larger than average	0.948	0.090	0.808***	12.667	5.882	6.755***
	Less than the mean	0.538	0.082	0.462**	9.590	4.673	4.551**
	Difference	0.409	0.008	0.402	3.077	1.210	1.868

Note: This table shows the results based on the nearest neighbor matching (1 to 5 matching) method, and the same procedure is used for the four matching methods using nearest neighbor matching (1 to 10 matching), radius matching, kernel matching (with a bandwidth of 0.06) and local linear regression matching.

***

**

And

* denote 1%, 5%, and 10% significance levels, respectively.

Thus, the estimated results on the impact of capital endowment differences on the size of credit access for farm households, the higher the endowment of physical, human, and social capital, the higher the key to digital credit and the total size of credit for farm households. Although more and more farm households are enjoying the benefits of digital technology, it cannot be ignored that there may also be a new divide within the rural population, which makes the financial well-being accessible to farm households with different capital endowments vary significantly.

## V. Conclusions and recommendations

This paper examines the impact of rural e-commerce development on rural households’ digital credit behavior at the micro-level using a multivariate Probit model and propensity score matching method with rural residents in the China Family Panel Studies (CFPS) database. Specifically, we examine the complementary or substitution relationship between rural digital credit and traditional bank credit and the impact of participation in e-commerce on the scale of rural digital credit. First, the empirical results show that there is a significant substitution relationship between digital credit and traditional bank credit; Second, the development of rural e-commerce not only expands sales channels, increases the scale of operation, and promotes farmers’ income, but also transforms individuals’ digital footprints in e-commerce platforms into credit data, completes credit accumulation, and further improves farmers’ financial welfare, thus forming a virtuous circle. Using propensity score matching method analysis, this paper finds that participation in rural e-commerce facilitates farmers’ access to more digital credit support. The total size of credit they receive increases substantially. Relative to those farmers who did not participate in e-commerce, those who participated in e-commerce increased their access to digital credit size and total credit size by $0.922 million and $37.49 million, respectively. Third, the differences in capital endowments of farmers lead to significant differences in the financial welfare obtained by farmers participating in e-commerce; those with high physical capital, increased human capital, and increased social capital has higher access to the scale of digital credit total credit size. The differences in capital endowments amplify the digital divide’s impact on farmers’ economic welfare effect.

Based on the above analysis, the following policy insights can be obtained from this paper. First, the development of digital credit leads to more significant pressure and challenges for traditional bank credit operations. In this regard, commercial banks should overcome their shortcomings, change their development mindset, improve their financial technology-based capabilities, actively carry out digital construction, accelerate the construction of digital banks and innovative banks, and pay attention to the credit needs of long-tail groups. Second, the lack of information literacy and financial literacy among farmers is an essential reason for the low percentage of digital credit usage. Therefore, the government should strengthen the promotion of digital technology in rural areas, popularize financial education, raise the scientific cognitive level of farmers about digital credit, reduce the behavioral bias of farmers in the process of digital credit selection, and further improve the universality and inclusiveness of digital finance development in rural areas. Third, the government should formulate a scientific and reasonable development plan for rural e-commerce, tailor it to local conditions, and bring into play the demonstration effect of typical local enterprises and capable people in villages to further tap the vast digital potential of rural e-commerce. Fourth, in promoting rural e-commerce development, special attention should be paid to the digital capabilities and literacy of rural "disadvantaged" groups, which will help them complete credit accumulation and alleviate financial constraints.

## Supporting information

S1 Dataset (XLS)
